# Public Health Interventions to Address Housing and Mental Health amongst Migrants from Culturally and Linguistically Diverse Backgrounds Living in High-Income Countries: A Scoping Review

**DOI:** 10.3390/ijerph192416946

**Published:** 2022-12-16

**Authors:** Gemma Crawford, Elizabeth Connor, Kahlia McCausland, Karina Reeves, Krysten Blackford

**Affiliations:** 1 Collaboration for Evidence, Research and Impact in Public Health, School of Population Health, Curtin University, Perth, WA 6845, Australia; 2 School of Population Health, Curtin University, Perth, WA 6845, Australia

**Keywords:** homelessness, housing, cultural and linguistic diversity, migration, mental health

## Abstract

Migrants from culturally and linguistically diverse (CaLD) backgrounds experience factors that may increase health inequities related to a range of determinants of health including housing and mental health. However, the intersection between mental health and housing for migrants is poorly understood. A scoping review searched four academic databases for concepts related to cultural and linguistic diversity, housing conditions, and public health interventions to address homelessness. A total of 49 articles were included and seven key themes identified: housing provision; mental health intersections and interventions; complexity and needs beyond housing; substance use; service provider and policy issues; the role of cultural and linguistic diversity; and consumer experience. The intersection of ethnicity with other social determinants of health and housing was highlighted though there were limited interventions tailored for migrants. Studies generally pointed to the positive impacts of Housing First. Other sub-themes emerged: social connection and community; shame, stigma, and discrimination; health and support requirements; and employment, financial assistance, and income. Consumer choice was identified as vital, along with the need for systemic anti-racism work and interventions. To support secure housing for migrants and mitigate mental health impacts, closer attention is required towards migration factors along with broader, tailored services complementing housing provision.

## 1. Introduction

Public health is confronted by issues inexorably linked to population mobility and migration, which can place individuals in situations that affect their physical and mental wellbeing and socioeconomic outcomes, including housing insecurity [1]. People on the move account for more than 280 million people worldwide, 3.6 percent of the global population [2]. Migration is recognized as a determinant of health and may exacerbate existing risk factors, increase individual vulnerability to the acquisition of infectious and non-communicable conditions and increase health disparities [1,2,3,4,5]. Influencing factors include the level of control over travel, experiences of trauma, transnational health practices, individual and organizational health literacy, cultural and linguistic diversity, migration and health policy, racism and discrimination, social networks, and support in country of origin and destination [2,6,7,8,9]. The International Organization for Migration (IOM) [10] has suggested that health has been marginal in the mobility and migration discourse, in part due to the cross-cutting nature of “health and migration” issues and determinants [11]. As Wickramage et al. [5] have argued, a limited understanding of the bi-directional relationship between migration and health has resulted in inadequate research, policy and practice activities in the intersection between these domains.

The flow of migrants from low and middle-income countries to high-income countries in North America, Europe, and Oceania is significant [3]. For example, more than half of Australians (51.5%) were either born overseas or have a parent born overseas [12]. Western Australia (WA) has the greatest proportion of overseas-born residents (35%), more than any other state or territory, with over 36,000 non-citizen arrivals in 2018 [13]. Migrants from culturally and linguistically diverse (CaLD) backgrounds settling in high-income countries (such as Australia) may experience a range of conditions pre, during, or post-movement, which amplify vulnerability to health inequities. Experiences before migration [14,15], such as political insecurity, family separation [8,16], trauma [17], and lack of adequate accommodation, education, and social stability [16] may increase mental health risks. Further, migrants from CaLD backgrounds may also experience disparities in housing, conceptualized as an essential social determinant of health [18].

Factors relating to housing security, such as homelessness, affordability, availability, and condition, affect physical and mental health outcomes [19,20,21,22]. Post-migration factors such as language barriers, employment difficulties, lack of income, lack of academic recognition or opportunities, and experiences of racism and discrimination [7,8,9,23] can increase the risk of mental health challenges and housing stress. Cultural expectations, sustained impacts of trauma, and marginalization can also exacerbate the risks of homelessness [6,9,14,24]. In turn, people who experience homelessness or housing stress are often at risk of a range of mental and physical health issues, social exclusion, and discrimination [7].

The intersecting housing and health needs of migrants from CaLD backgrounds are not well understood in the Australian context. Data from 2016 showed that 116,000 people in Australia experienced homelessness, and 15% of people born overseas or who had arrived in Australia within the previous five years were estimated to be homeless [25]. Around three-quarters (74%) of those born overseas/arrived in Australia within five years lived in severely crowded dwellings and 13% lived in boarding houses [8,26,27]. WA has the highest proportion of people born overseas nationally (32%); on census night in 2016, 12.7% of people who accessed homelessness services were born overseas [25]. Although these data provide some insights, it is worth noting that homelessness data in the census are likely underreported, as rough sleepers are often not adequately captured.

Despite these statistics, there is a lack of published research addressing effective interventions for homelessness and housing [23] and people from CaLD backgrounds are noticeably absent in strategies and frameworks to address housing and mental health [28,29]. Further, intervention and service evaluation indicate they may not meet the needs of refugees and migrants [7,14,30]. For example, findings from Multicultural Mental Health Australia show that people from CaLD backgrounds with a mental illness are at increased risk of long-term homelessness due to a lack of accessibility to appropriate services and resources [31]. Other data have suggested that while housing and social services provided by the Australian Government are more likely to be successful in securing long-term tenancies for refugees, around 25% experience housing affordability issues following settlement [32] and less than 10% of refugees access public housing services within 18 months of resettling in Australia [33].

To build the evidence base for effective public health interventions and a greater focus on health and housing in the context of migration, a scoping review was conducted to explore existing interventions and evidence related to homelessness, housing, and mental health, concerning migrants from CaLD backgrounds.

## 2. Methods

### 2.1. General Approach

The Joanna Briggs Institute Methodology for Scoping Reviews [34], PRISMA Extension for Scoping Reviews (PRISMA-ScR) [35], and the Arksey and O’Malley [36] methodological framework of scoping studies guided the scoping review. The review used the following steps: (1) identifying the research question; (2) identifying relevant studies; (3) study selection; (4) charting the data; (5) collating, summarizing, and reporting the results; and (6) consultation to access expert knowledge to inform and validate findings.

#### 2.1.1. Identify the Research Question

The review applied an expansive understanding of homelessness as more than just “rooflessness” [37] to capture a range of experiences, including lack of suitable accommodation, inadequate housing conditions, unstable tenure, and lack of space for social interactions [38]. Broader notions of housing precarity, such as overcrowding, were also included [27]. Definitions included those from the Australian Institute of Health and Welfare (AIHW) that suggest homelessness occurs when an individual’s current housing circumstance “is in a dwelling that is inadequate; has no tenure, or if their initial tenure is short and not extendable; or does not allow them to have control of and access to space for social relations” [39]. In addition, a widely accepted “cultural” definition of homelessness was drawn upon, proposed by Chamberlain & MacKenzie [40], that captures concepts of primary homelessness, constituting a lack of “conventional accommodation” in addition to secondary and tertiary homelessness, which encompass transient accommodation arrangements, living with others or in emergency accommodation, and living in single rooms without private amenities or housing security. These definitions are important, as evidence shows that people from CaLD backgrounds may experience secondary and tertiary homelessness more commonly than primary homelessness [27]. Additionally, there is evidence that cultural understandings of, and approaches to housing may include practices that would constitute unstable housing arrangements and indicate vulnerability for homelessness, such as acceptance of overcrowding as reasonable in the context of cultural obligations and avoiding homelessness [6,27,41].

Definitions of “culturally and linguistically diverse” vary. The term is contentious with the potential to suggest homogeneity of experience and an “assumption of vulnerability” [27]. The Office of Multicultural Interests [42] in Australia defines CaLD as “the wide range of cultural groups and individuals that make up the Australian population. It includes groups and individuals who differ according to religion, race, language, and ethnicity except those whose ancestry is Anglo-Saxon, Anglo-Celtic, Aboriginal or Torres Strait Islander”. Kaleveld et al. [27] refined this definition to capture factors relevant to social vulnerability, including: “being born in a country other than Australia that is Non-Anglo/Celtic; and, speaking languages other than English, and/or not speaking English well; and recency of arrival (people who have been in Australia less than five years are more vulnerable)”. This is the broad definition applied in the present study to provide some parameters to the search whilst recognizing the contested nature of the terminology.

Social determinants of health, such as education, employment, and income, have a significant impact on housing experiences and mental health outcomes for migrants from CaLD backgrounds [9,19,20]. The literature calls for comprehensive public health approaches to housing and homelessness that address the social determinants of health [43]. Therefore, this review focused on public health interventions that addressed social determinants of health or applied universal (e.g., interventions to improve outcomes for an overall population), primary prevention (e.g., interventions designed to prevent poor health or social outcomes before they occur), or early intervention (e.g., interventions targeting people experiencing early signs and symptoms of health or social problems) approaches [44]. Approaches focused on highly individualized or exclusively clinical interventions, such as medications or other therapies without complementary, holistic preventative approaches, were excluded.

Studies conducted in high-income countries with forms of universal healthcare [45] were included, to understand interventions implemented in socio-economically comparable settings to Australia. High-income countries have a Gross National Income per capita of USD 12,376 or more [46]. The review included the United States of America (USA) due to its high-income status, and the availability of some forms of universal health care, including Medicare and Medicaid, for specific populations [47].

An initial search for existing reviews published between 2000 and 2020 was undertaken. The initial search did not identify existing systematic reviews or meta-analyses focused on homelessness, mental health, and people from CaLD backgrounds. Preliminary research and definitions refined the review scope and inclusion criteria for studies (Table 1). The final study outcomes were to (1) map the current literature base on this topic area; (2) identify the key themes in the literature on interventions concerning housing, homelessness, and mental health for people from CaLD backgrounds; and (3) identify gaps in the research area.

#### 2.1.2. Identify Relevant Studies

Studies were identified through four databases selected through recommendation by the University librarian: Proquest, PubMed, Scopus, and PsychInfo. Specific medical subject headings (MeSH) terms were employed (Table 2). 

#### 2.1.3. Select Studies

Identified articles (n = 3582) were extracted into Endnote version X9 [48]. Article titles and abstracts were screened by two reviewers, excluding any articles that did not meet the eligibility criteria. Then, two reviewers briefly assessed eligible articles for relevance and scope. The full text of each remaining article was then reviewed to determine the final set of articles for inclusion, excluding eligible articles. The reference lists of all included literature were hand-searched to identify relevant literature not indexed in the electronic databases. This process continued until no new results were found. Three reviewers were involved in this process; two lead reviewers checked the results to minimize errors. A third reviewer provided guidance where reviewers could not determine an article’s eligibility and analyzed approximately 25 percent of the articles retrieved to ensure consistent application of the inclusion criteria. A consensus panel of the research team mediated reviewer disagreements. The research team comprised early and mid-career academics and practitioners working in public health with experience in migration, mental health, and housing.

#### 2.1.4. Chart the Data

A descriptive-numerical approach was used to chart article data [49]. The data extraction table was initially developed based on the results of the preliminary scoping phase and the Arksey and O’Malley [36] scoping study methods. The table was refined based on a review of included studies and by identifying recurring features of interest as data extraction proceeded. In the final iteration, data from the included studies (n = 49) were extracted into a table (see Table S1). 

#### 2.1.5. Collate and Summarize the Data

Data table content was collated using Microsoft Word 365, and Microsoft Excel was used to record and summarize key numerical elements of the data analysis. Following the development of the data table, key content for further description was transferred into a word document, and narrative descriptions were developed. An inductive approach to thematic analysis was used to explore studies, consistent with the approach of Khan et al. [50]. Extracts were identified from each article that demonstrated emerging themes. These findings were grouped into larger, overarching themes, creating a set of themes and sub-themes. Themes and sub-themes were refined and reviewed during consultation (stage 6).

#### 2.1.6. Consultation

Key stakeholders were consulted to refine themes and identify additional, relevant literature. Members of a research steering group comprising senior representatives from housing services, mental health organizations, social services, and services working with people from CaLD backgrounds provided input. Initial data tables, themes, and narrative descriptions were sent to experts, accompanied by a survey developed and disseminated via Qualtrics XM [51]. The survey content was adapted from Khan et al. [50] and asked participants to assess the data table, themes, and narrative analysis based on their knowledge and expertise. Dichotomous questions with binary answers were included, which related to the accuracy of themes, missing themes, additional literature, and the relevance of themes to the Australian context. Findings indicated that themes were accurate, no themes had been missed, and no critical pieces of relevant literature had been overlooked. Themes and sub-themes were considered relevant to the Australian context. Results informed the final iteration of themes and narratives.

## 3. Results

### 3.1. Study Characteristics

**Figure 1:**
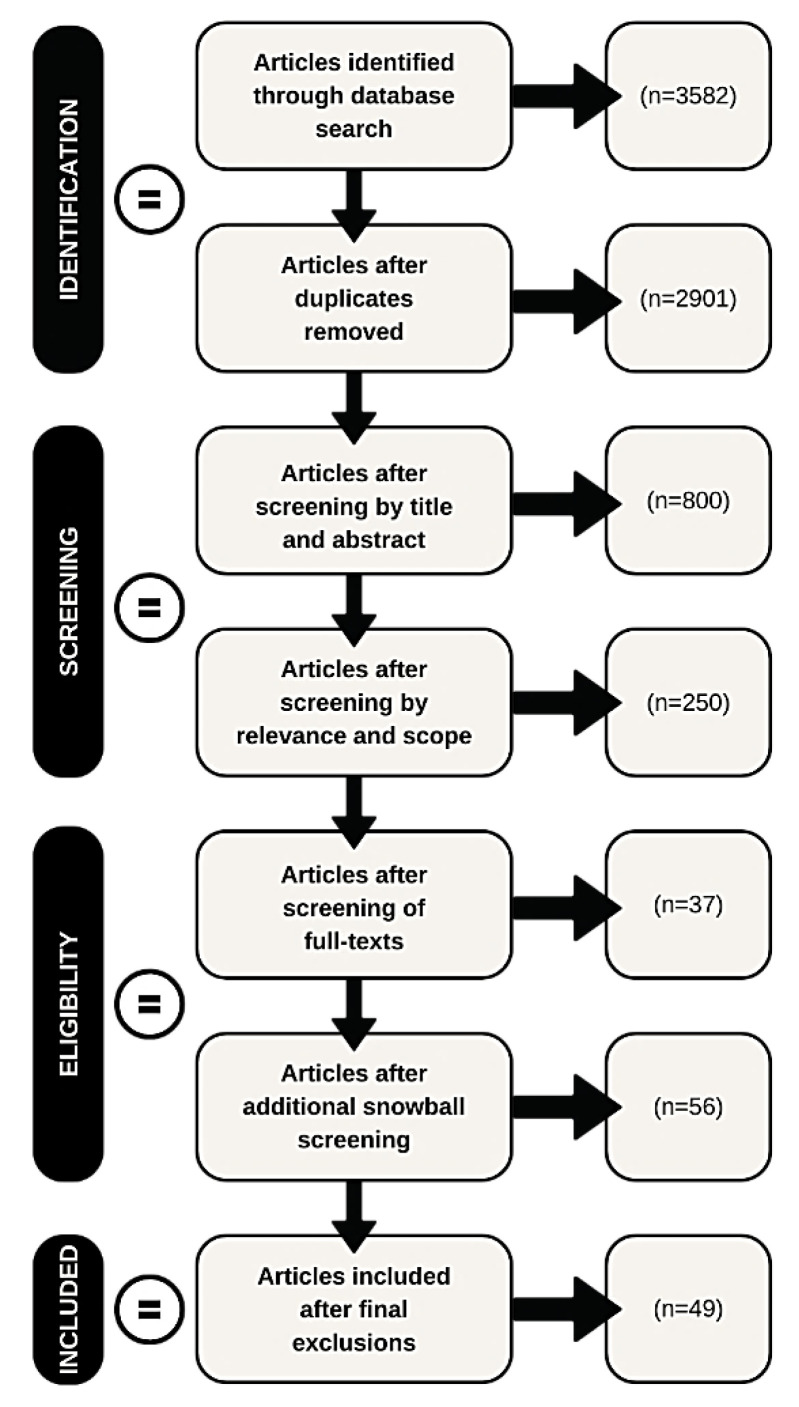
Selection of studies.

A total of 49 articles were included in the final review. The PRISMA Extension for Scoping Reviews (PRISMA-ScR) [35] flowchart for the selection of sources of evidence (Figure 1) demonstrates the selection process and final article numbers for this review. A summary of extracted data can be found in Table S1.

The study sample size ranged significantly from 20 [52] to 853,012 [53] participants. A majority of studies were published from 2010 onwards; around one in five studies (22.4%, n = 11) were published prior [54,55,56,57,58,59,60,61,62,63,64]. The study location was limited to three countries, with around half based in the USA (51%, n = 25) [53,55,56,57,59,60,61,62,63,64,65,66,67,68,69,70,71,72,73,74,75,76,77,78,79], 40.8% (n = 20) in Canada [58,80,81,82,83,84,85,86,87,88,89,90,91,92,93,94,95,96,97,98,99], and 6.1% (n = 3) in the United Kingdom (UK) [52,54,100]. Studies were predominantly quantitative (83.7%, n = 41). More than half the studies were randomized control trials (57.1%, n = 28). See Table 3 below for a summary of the study designs and corresponding citations.

### 3.2. Intervention Types and Outcomes

Of the main categories of intervention, around three-quarters (73.5%, n = 36) provided housing and shelter provision-related interventions, while 10.2% (n = 5) provided physical health-related interventions, including substance use interventions. Table 4 provides further detail about intervention types and corresponding citations.

Housing First is an international model (see Table 5) to reconceptualise responses to homelessness by providing safe and permanent housing as the first priority [101].

### 3.3. Reporting of Cultural and Linguistic Diversity

Reporting of cultural and linguistic diversity was varied. All articles referenced ethnicity, cultural background, race, or related identities. Less than a quarter of studies (20.4%, n = 10) specifically addressed outcomes or insights related to CaLD status [52,54,58,69,77,78,86,93,95,97]. Only 14.3% (n = 7) of the studies were purposefully designed to explore or address the needs of people from CaLD backgrounds (as opposed to reporting on outcomes against cultural and linguistic diversity) [52,54,58,77,93,95,97]. Table 6 provides an overview of the cultural and linguistic classifications used by studies and corresponding citations.

### 3.4. Themes and Sub-Themes

Seven themes and 18 sub-themes were identified (Table 7), discussed below.

### 3.5. Housing Provision

Most studies (see Table 4) explored the impact and sustainability of housing provision for people experiencing homelessness and mental health issues. Three sub-themes emerged: Housing First—efficacy and considerations; other housing provision models; and costs of housing provision.

#### 3.5.1. Housing First—Efficacy and Considerations

Most studies focused on Housing First interventions. Two studies explored the recruitment processes for Housing First interventions [84,91], finding that a diverse range of participants recruited were commonly affected by homelessness with mental and physical illnesses. Housing First interventions were reported as effective in achieving improvements across various domains for people experiencing homelessness and mental health challenges: housing stability [56,61,63,82,83,87,93,94,95,96]; speed of housing attainment [63,83]; quality of life [81,83,87,89]; levels of mental health and substance use recovery [83]; psychiatric hospitalizations and mental health [87]; and community functioning [81]. Several studies noted the efficacy of Housing First approaches for people with a broad range of needs including substance use [88,98], and across ethno-racial groups [97], with the universal application of Housing First approaches supported [62,65,80,99]. However, some studies noted that improvements in measures such as quality of life, community functioning, and social integration for migrants from CaLD backgrounds were either not achieved [93,94] or not sustained long-term [96]. In some studies, Housing First was found to have no impact on substance use [59,85,92].

#### 3.5.2. Other Housing Provision Models

In addition to Housing First, a range of other housing models were explored, many of which were based on the premise of permanent housing [75,78], and supported housing approaches based on Housing First principles [55,67,68,69,72], or mixed models [60]. These models demonstrated the centrality of housing provision to reducing homelessness [60] and were shown to lead to improvements in housing stability [75]; reductions in experiences of stigma and discrimination [78]; and positive evaluations of housing arrangements when people were rehoused [100]. Of note, two studies [66,67] found that health service use increased in the context of Full-Service Partnership models (defined by the authors as “Housing First programs that do “whatever it takes” to improve residential stability and mental health outcomes”), while one study [75] found that Permanent Supportive Housing (in line with Housing First principles) was not effective in increasing social engagement and inclusion among migrants from CaLD backgrounds.

#### 3.5.3. Costs of Housing Models

Housing First and other housing models were consistently found to be cost-effective and were either cheaper than alternative models [56,61] or offset a significant amount of the intervention’s cost [66,82]. For example, Aubry et al. [82] found that Housing First produced a 96% cost offset, while Gilmer et al. [66] found that Full-Service Partnerships offset 82% of their cost. Similarly, [94] found that the cost associated with Housing First with Intensive Case Management (where participants work with a case manager to develop an individualized service plan based on goals for recovery) was almost a third less than that of supportive housing with Active Community Treatment, with fewer instances of emergency shelter utilization and reductions in the use of single-room accommodation options accounting for much of this offset. However, Gilmer et al. [67] noted that outpatient mental health visits increased with the provision of Full-Service Partnership housing interventions, with associated costs.

#### 3.5.4. Mental Health—Intersections and Interventions

Consistent with the review objectives, the mental health of migrants from CaLD backgrounds experiencing or vulnerable to homelessness was a key theme. In many studies, mental health status was a selection criterion for participants, especially in the context of Housing First interventions [63,82,83,87,93,94,95,96]. However, beyond this logistical consideration, a range of sub-themes relating to the intersection of mental health, housing, homelessness, and the impact of non-housing-based interventions emerged.

#### 3.5.5. Housing, Homelessness, and Mental Health

Intersections between mental health, homelessness, and housing were key considerations. Several themes emerged, including that mental health status did not place people at significant risk of returning to homelessness or compromise housing outcomes in housing provision programs [65]. Findings also suggested that housing provision reduced the risk of homelessness for people with mental health challenges [60] and that mental health diagnoses were generally not implicated in reduced housing stability with the appropriate support [81,95].

Results were mixed regarding the impact of housing-based interventions on mental health outcomes. Several studies noted that housing interventions did not improve mental health outcomes compared to usual treatment [63,68,81,82,93,100]. Other studies found improvements in mental health outcomes with housing interventions [75,87], while Karim et al. [100] and Aubry et al. [83] found perceived improvements in mental health and recovery in the context of housing. Some studies found that housing provision interventions increased the utilization of mental health outpatient services [66,67] while others found the opposite [59].

#### 3.5.6. Mental Health Interventions for People Experiencing Homelessness (Non-Housing)

There were inconsistent results for non-housing-based interventions for mental health among people who were homeless. Schwan et al. [90] noted the potential of arts-based programs for addressing mental health for young people experiencing homelessness, and Walter et al. [77] found reductions in depressive and anxiety symptoms because of a health center-based model for Latinx adults. Schueller et al. [71] found no change in mental health symptoms with a mobile phone-based intervention (but a high level of acceptability), and Winiarski et al. [79] found no change with a health center-based model for young people. Similarly, Slesnick et al. [73] found no difference in outcomes between three mental health interventions with young people experiencing homelessness, although symptoms improved in all treatment conditions.

#### 3.5.7. Complexity and Needs beyond Housing

While the provision of housing was a focus of most studies (see Table 4), non-housing-related needs in the context of homelessness and mental health were also identified. Sub-themes included social connection and community; shame, stigma, and discrimination; health and support requirements of people who are homeless; and employment, financial assistance, and income support.

#### 3.5.8. Social Connection and Community

While Harris et al. [69] identified consistently low rates of social support and networks in populations vulnerable to homelessness, other studies highlighted the role and value of social connection and community in the context of housing, homelessness, and mental health. For example, social connections, including family and friends, were important in providing housing and informal sources of information about housing options for migrant and refugee populations at risk of homelessness [54,58]. Similarly, the value of group-based activities for addressing mental health in people who are homeless, including people from CaLD backgrounds, was noted by Thompson et al. [52] and Travis et al. [74].

#### 3.5.9. Health and Support Requirements of People Who Are Homeless

Studies suggested a high prevalence of health needs for people experiencing homelessness [91], with health and support service needs as significant considerations [68,100]. The role of health and other support services was identified as a support for families at risk of homelessness [57,68] and health-based interventions (specifically related to sexual health and alcohol and other drug use) were demonstrated to be effective in populations vulnerable to or experiencing homelessness [64,76]. Importantly, studies indicated a central need for health services, social support, and recovery-oriented support to complement housing interventions [55,96].

#### 3.5.10. Shame, Stigma, and Discrimination

The literature highlighted experiences of shame, stigma, and discrimination for migrants from CaLD backgrounds at risk of or experiencing homelessness and mental health challenges [52,64,86]. For example, Mejia-Lancheros et al. [86] identified that adults experiencing homelessness and mental illness faced discrimination in healthcare settings, while Thompson et al. [52] also found that migrants from CaLD backgrounds in the UK experienced shame when asking for assistance with housing. Conversely, Wenzel et al. [64] found that permanent supportive housing provision reduced everyday discrimination against people previously experiencing homelessness.

#### 3.5.11. Employment, Financial Assistance, and Income Support

Financial and employment issues were reported in several studies. Thompson et al. [52] noted employment as a key issue for men from CaLD backgrounds in the context of homelessness. Karim et al. [100] found that families experiencing homelessness wanted more information about work and financial issues. Pilkauskas and Michelmore [53] found that financial assistance via income support programs improved housing outcomes, including overcrowding and doubling up on living arrangements.

#### 3.5.12. Substance Use

Substance use was a recurring theme. In particular, the relationship between housing and substance use was evident, and exploring substance-use interventions in the non-housing space was a key focus.

#### 3.5.13. Housing Provision and Substance Use

The literature addressed substance use outcomes in the context of housing interventions and mental health. Early work by Tsemberis and Eisenberg [62] found that dual diagnosis with mental health and substance use reduced housing security. However, multiple studies [72,77], and specifically in the context of Housing First interventions [62,88,98], suggested that substance use did not significantly or negatively impact on housing outcomes and did not predict a return to homelessness [65]. While Padgett et al. [70] found secure housing (Housing First) supported reductions in substance use, there was more evidence that housing provision in the context of Housing First interventions was not effective in reducing substance use compared to usual treatment [85,92], including among people from CaLD backgrounds [93]. Slesnick and Erdem [72] noted a lack of evidence regarding whether housing alone was sufficient to reduce alcohol and substance use or whether targeted interventions were required.

#### 3.5.14. Substance Use Interventions (Non-Housing)

A small number of studies explored non-housing-related interventions for substance use, with mixed results. Slesnick et al. [73] found a variety of interventions for substance use delivered in shelter settings were effective in reducing substance use, although overall use remained high. Hanrahan et al. [57] and Walter et al. [77] explored healthcare setting-based models. For example, the study by Walter et al. [77] was conducted with Latinx populations to examine the impact of integrated and co-located behavioral and primary health care delivered via a multilingual and multicultural substance use and mental health residential treatment service. The study observed short- and long-term changes in substance use (reduction in illicit drug use). Hanrahan et al. [57] conducted a pilot intervention offering psychosocial rehabilitation and intensive care management to mothers with psychiatric illnesses who were homeless. The study found six- and 12-month reductions in substance use.

#### 3.5.15. Service Provider and Policy Issues

A recurring theme was the importance of service providers and policy-based issues in the context of housing, homelessness, mental health, and cultural and linguistic diversity.

#### 3.5.16. System Change

Of interest, several studies identified the need for system-level change to adequately address the needs of populations vulnerable to homelessness or mental health issues in the context of cultural and linguistic diversity. Dwyer and Brown [54] noted the need for broader, systemic anti-racism work to reduce housing insecurity risk among migrants, while Mejia-Lancheros et al. [86] advocated for broader systemic policy and education interventions to decrease stigma and discrimination around mental health, alcohol and other drug use, and homelessness. Stefanic and Tsemberis [61] noted the need for a broadened scope regarding who could be appropriately housed and a shift in focus from providing alcohol and other drug services to housing for vulnerable populations. O’Campo et al. [87] identified the need for better integration between the housing and health sectors. Karim et al. [100] similarly identified the need for a coordinated strategy to address complex, non-housing needs in families who were homeless and vulnerable to mental health challenges. Stergiopoulos et al. [97] identified the role of Housing First services in advocacy for systemic reform (e.g., anti-racist and other approaches to support community engagement and mobilization) and to address the requirements of people from CaLD backgrounds.

#### 3.5.17. Service Needs and Gaps

The need for resourcing, training, and adequate investment for housing and homelessness services was identified [90], including the need for greater awareness about staffing and resourcing considerations in the context of Housing First initiatives and the need to engage and educate communities in developing and implementing new services [61]. Several gaps in service delivery for populations vulnerable to homelessness were also identified, including a lack of formal housing assistance for refugee populations [58], inadequate housing provision, and a lack of coordination between service providers and sectors [54]. The role of community organizations in supporting migrant populations was identified, but lack of funding and consequent transience of service provision were cited as barriers to effectiveness [54].

#### 3.5.18. The Role of Cultural and Linguistic Diversity

All studies collected data on cultural and linguistic diversity or ethnicity; however, few explicitly focused on outcomes related to these identifiers. The intersection of ethnicity with other social determinants of health and housing was a recurring theme, as was the value of tailored approaches for people from CaLD backgrounds.

#### 3.5.19. The Intersection of Ethnicity

The intersection of ethnicity and housing, homelessness, and mental health was identified. For example, Dwyer and Brown et al. [54] noted that forced migration increased vulnerability for homelessness, with inadequate policy and service provision to cater to those in this group. Similarly, Murdie [58] observed that refugee status impacted housing trajectory, with refugee claimants more vulnerable to housing instability and issues such as housing affordability. Mejia-Lancheros et al. [86] noted an interaction between ethno-racial status, mental health, and experiences of stigma, while Wenzel et al. [78] reported the pervasive nature of societal discrimination and racism on outcomes for marginalized populations. Few studies noted positive or neutral effects for people from CaLD backgrounds in housing and homelessness outcomes. Schutt et al. [60] found that ethnicity had no impact on housing stability, while Stergiopoulos et al. [93] found that greater improvements were seen in participants from CaLD backgrounds on all measures in a Housing First study. Similarly, Harris et al. [69] found that participants vulnerable to homelessness who identified as Black had more robust social support networks than other groups in their research.

#### 3.5.20. The Value of Tailored Approaches for People from CaLD Backgrounds

Several studies identified the value of tailored approaches for people from CaLD backgrounds. Stergiopoulos et al. [97] found that holistic treatment provided as part of tailored approaches for people from CaLD backgrounds with anti-racism and anti-oppression principles was valued by service users, including addressing the social and cultural determinants of health. Similarly, Stergiopoulos et al. [95] found that Housing First augmented by anti-racism and anti-oppression approaches increased housing stability and community integration for people from CaLD backgrounds. However, no changes in mental health outcomes were observed. Walter et al. [96] also found that culturally responsive treatment and support systems improved wellbeing and long-term recovery for Latinx adults with mental health challenges vulnerable to homelessness.

#### 3.5.21. Consumer Experience

Studies identified the importance of core components of the consumer experience in the context of housing, homelessness, and mental health, including the value of working with people with lived experience, consumer choice, and novel ways of working with people experiencing homelessness.

#### 3.5.22. Working with People with Lived Experience

The value of lived experience in shaping and guiding intervention design and implementation was noted by several studies [64,76,84,90]. Such perspectives were identified as key to ensuring the accuracy and relevance of interventions to research participants. For example, Winiarski et al. [79] identified tensions between the views of service providers compared to those of young people receiving mental health interventions in a shelter-based setting, highlighting a need for more effective consultation with young people with lived experience to ensure the efficacy of and engagement with services.

#### 3.5.23. Consumer Choice

Consumer choice was identified as vital, with implications for consumer satisfaction and intervention efficacy. Gulcur et al. [56], and Tsemberis et al. [63] noted that choice was a key component of Housing First interventions, with Gulcur et al. [56] positing that choice may contribute to the success of housing retention in these models. Aubry et al. [83] noted that the choice of housing options was linked to enhanced satisfaction with housing arrangements. Similarly, Padgett et al. [59] suggested that engagement and retention in Housing First models may be enhanced when clients with mental health and substance use diagnoses are provided with the option of actively participating in their treatment decisions. Tsai et al. [75] also noted the important roles of consumer choice and empowerment in recovery and social integration for people experiencing homelessness and mental illness.

#### 3.5.24. Novel Ways of Engaging People Vulnerable to or Experiencing Homelessness

Schwan et al. [90] and Travis et al. [74] identified the benefits of art and hip-hop-based creative therapies for improving the mental health of people experiencing homelessness. Schueller et al. [71] found that mobile phone-based interventions were feasible and acceptable to engage young people experiencing homelessness, although improvements in mental health outcomes were not achieved. Similarly, Winiarski et al. [79] noted the potential of technology-based interventions to engage young people experiencing homelessness and mental health challenges, to enhance engagement with mental health interventions.

## 4. Discussion

People from CaLD backgrounds experience unique and complex factors concerning vulnerability to homelessness and mental health [23,27,32]. Comprehensive public health approaches to housing and homelessness that address the social determinants of health are required [43]; however, there is a lack of evidence about effective interventions for homelessness and housing for people from CaLD background in Australia [23], including migrants. This scoping review explored the literature regarding public health approaches to housing, mental health, and migration. While the intersection of ethnicity and housing, homelessness, and mental health was identified throughout the review, results indicated very few studies were explicitly designed to address the needs of migrants from CaLD backgrounds [52,54,58,78,93,95,97]. Themes highlighted significant complexity regarding housing provision, intersections with mental health and substance use, social determinants of health beyond housing, structural issues and consumer experience, and the role of cultural and linguistic diversity. Findings are discussed below in the context of the broader literature.

### 4.1. Housing Is Key but Services to Complement Housing Are Required

Housing provision to support people with mental health challenges who are vulnerable to homelessness is critical. A significant proportion of the studies explored Housing First initiatives or models based on this approach. Housing First offers a model which provides the dignity of housing regardless of other health comorbidities and is cost-effective [56,61]. Studies showed that housing stability could be achieved regardless of substance use [72,77,88] and mental health status [65,81,95]. However, these models did not reliably increase social inclusion and integration [93,94,96], and evidence for the effectiveness of non-housing-related interventions for substance use and mental health was inconsistent [57,71,73,79]. These findings are of particular importance concerning another emerging theme: the complexity and breadth of non-housing needs experienced by people vulnerable to homelessness and mental health challenges, including migrants from CaLD backgrounds. Other research echoes these findings and calls for improved public health responses to address a range of needs for refugees and other migrants after resettlement [105], including services that complement Housing First approaches and foster social inclusion [106]. However, published research is lacking in detailing the drivers and experiences of these needs or how best to address them. This paucity of evidence reinforces the importance of effective and sustained public health approaches to homelessness and mental health [43].

### 4.2. Greater Attention Is Needed toward Indicators of Cultural and Linguistic Identity and Diversity

While ethnicity, and race and ethnicity, were used most often to describe differences in identity, categorizations varied widely. Flatau et al. [32] have also identified inconsistencies in the definitions of CaLD in the context of epidemiological research which can compromise the quality, comparability, and generalizability of research. There are practical and policy implications for public health regarding applying research findings and identifying and addressing the needs of migrants from CaLD backgrounds. Addressing this lack of consistency in reporting is critical [23,27,32]. There are tensions in the literature regarding indicators of race, culture, and ethnicity; uses of which have been criticized for reifying differences in health outcomes experienced between groups [107] (Brady, 2012). The Federation of Ethnic Communities’ Councils of Australia (FECCA) has been vocal regarding perceived deficits in how cultural, ethnic, and linguistic diversity data are collected and reported [108]. Wickramage et al. [5] have argued for better research data that engage “complex, dynamic and often intersecting migrant typologies.” While a theme emerged regarding the intersection of ethnicity with housing and other social determinants of health, a minority of studies specifically addressed outcomes or insights related to CaLD status or were designed for people from CaLD backgrounds [52,54,58,69,77,78,86,93,95,97]. Other areas of health also observe a lack of reporting on the specific needs of people from CaLD backgrounds [109]. For example, Minas et al. [110] noted a ubiquitous lack of reporting in Australia on mental health data for people from CaLD backgrounds. Murray et al. [111] also noted the broad exclusion of people from CaLD backgrounds in Australian health research, a gap to be addressed. Future research should consider how inconsistent classification and reporting might be addressed and how outcomes for people from CaLD backgrounds can be prioritized. Input from people from CaLD backgrounds is required regarding appropriate and meaningful indicators and research studies.

### 4.3. Systemic Change Is Vital

The literature highlights the macro drivers and causes of homelessness, including migration as a structural risk factor [112]. Several studies called for systemic reform to address the requirements of people experiencing homelessness and mental health challenges, including people from CaLD backgrounds. Notably, the need for systemic anti-racism work [54] and interventions to reduce stigma and discrimination at a systemic level [86] were identified, reflecting the experiences of people from CaLD backgrounds in these areas. These calls reflect the growing recognition of racism as a public health issue in and of itself, with impacts on the social determinants of health, including housing and health outcomes for people from CaLD backgrounds and people from ethnic minority populations [113,114,115]. System- and organization-level initiatives to address racism have been proposed for health and public health, with strategies such as employing a social-justice lens, providing anti-racism education, increasing awareness of the consequence of racism, and engaging in political advocacy to affect change [114,115]. Approaches such as these can be employed by stakeholders working in the housing and mental health spaces to address racism and improve outcomes for people from CaLD backgrounds. The review highlighted the role of service providers, including community-based organizations, in supporting people from CaLD backgrounds with housing [54,58]. This finding reflects the importance of the role of community services in providing complementary support to housing provision for people from CaLD backgrounds, as identified in Australia [9], WA [29], and internationally [106]. Importantly, to develop or strengthen services and system change, consumer choice should be prioritized [64,83,90]. Further, a commitment to meaningfully engage people with lived experience and prioritize these perspectives in the development, implementation, and evaluation of housing and homelessness-related interventions should be pursued.

## 5. Strengths and Limitations

### 5.1. Study Design and Reporting Limitations

Most included papers cited methodological limitations (93.9%, n = 46) regarding research design, data collection or interpretation of results; three did not (54, 56, 62). Study authors reported a range of limitations including issues with variables or measures; study design issues, such as non-binding, non-randomization, and non-uniformity of interventions; self-reporting and bias issues; short follow-up time; lack of generalizability; sampling issues, including size, method, and lack of access to priority groups; and missing data, lack of supporting evidence, and data accuracy issues. Eleven studies [52,53,54,57,58,60,62,63,75,76,100] did not report ethical oversight, which may lead to methodological weakness or subject burden. There were few, specific, qualitative studies (n = 4) [54,58,70,74,90] limiting rich contextual insights into behavioral outcomes. As the results identified, inconsistency in terminology, definitions, and categorization concerning cultural and linguistic diversity presents a challenge for consistency and quality in measurement, implementation, and monitoring.

### 5.2. Strengths and Limitations of the Review

This review has several strengths. To our knowledge, it presents a unique snapshot of the peer-reviewed literature regarding homelessness, mental health, and cultural and linguistic diversity. Using multiple databases, search terms and variations over a 20-year timeframe provided expanded scope. Using multiple researchers to undertake database searching reduced any margin for error. Including qualitative and quantitative studies with varied study designs expanded the review’s scope. The methodology facilitated the extraction of broad themes of importance from the existing literature iteratively and inductively. Themes have the potential to guide future research in the field.

The review has several limitations. A rigorous and iterative search was employed to identify articles, and stakeholder consultation was utilized to ensure a broad range of literature was captured. However, it is possible that relevant literature may not have been identified. Only English language articles in a defined period were included; papers in other languages and outside the timeframe for inclusion may have identified additional relevant studies. The review focused on the academic literature, which meant that there may have been an inherent level of publication bias. Many countries not represented in the review may likely contribute additional valuable insights to our findings but have not contributed their findings to the peer-reviewed literature for various reasons.

## 6. Conclusions

Migrants from CaLD backgrounds experience unique factors concerning housing and mental health, requiring comprehensive public health approaches to address these needs. This scoping review revealed limited interventions tailored for migrants from CaLD backgrounds. To date, a large proportion of research about homelessness, mental health, and cultural and linguistic diversity has centered on the positive impacts of Housing First approaches. The literature revealed key themes concerning the complex needs of people vulnerable to homelessness, the intersection of cultural and linguistic diversity, and the need to engage genuinely with people with lived experience, with calls for systemic change to address these needs. Identified gaps in the knowledge base can guide future research, focusing on the specific needs and outcomes of migrants from CaLD backgrounds. Insights should guide services and supports required to complement housing provision and to improve broader outcomes for migrants from CaLD backgrounds vulnerable to or experiencing homelessness and other mental health challenges.

## Figures and Tables

**Table 1:** Inclusion criteria for studies.

Inclusion Criteria
Full text.
Quantitative and qualitative primary studies.
Peer-reviewed literature.
Published between 2000 and 2020.
English language.
Study intervention: *(1) Public health focus and/or address social factors that influence housing (determinants of housing); and* *(2) Include mental health outcomes such as changes in self-reported mental health, signs, and symptoms of mental health problems.*
Study population: *(1) People from CaLD backgrounds;* *(2) Over the age of 18 years;* *(3) Living in high-income countries;* *(4) Access to a form of basic universal health care; and* *(5) Vulnerable to homelessness.*

Studies were excluded that (1) focused on in-country migration; (2) were not published in English; and (3) were exclusively clinically focused.

**Table 2:** MeSH Search Terms.

Migrant	“Immigrant*” OR ethnic OR “Culturally and Linguistically Diverse” OR “Ethnic minority*” OR “migra*” OR “Asylum seeker” OR refugee*
Living condition	Homeless*
Interventions	intervention* OR strateg* OR “health education” OR “community services” OR “social services” OR housing OR shelter OR “primary health*” OR policy OR evaluat* OR outcome OR impact OR efficacy OR effectiveness

**Table 3:** Study design summary.

Study Type	No. of Studies (%)	Citation
Randomized control trial	28 (57.1)	[55,56,60,61,63,68,72,73,76,80,81,82,83,84,85,86,87,88,89,91,92,93,94,95,96,97,98,99]
Longitudinal study	6 (12.2)	[59,69,75,77,78,100]
Qualitative	5 (10.2)	[54,58,70,74,90]
Mixed methods	3 (6.1)	[52,64,71]
Quasi-experimental study	3 (6.1)	[53,66,67]
Retrospective chart review	1 (2.0)	[57]
Comparative study	1 (2.0)	[62]
Cross-sectional evaluation	1 (2.0)	[79]
Non-randomized control trial	1 (2.0)	[65]
**TOTAL**	**49 (100)**	

**Table 4:** Interventions.

Intervention Type	Program	No. of Studies (%)	Citation
**Housing and shelter provision**	Housing first	26 (53.0)	[56,59,61,62,63,70,77,80,81,82,83,84,85,86,87,88,89,91,92,93,94,95,96,97,98,99]
	Full-service partnership	2 (4.1)	[66,67]
	Ecologically based treatment	2 (4.1)	[68,72]
	Permanent supportive housing	2 (4.1)	[69,78]
	Supportive housing	1 (2.0)	[55]
	Rehousing in the community	1 (2.0)	[100]
	Housing—various	1 (2.0)	[60]
	Collaborative initiative to help end homelessness	1 (2.0)	[75]
	**Total**	**36 (73.5)**	
**Physical health including substance use**	Thresholds mothers project	1 (2.0)	[57]
	Substance use interventions	1 (2.0)	[73]
	AWARE: AOD and health program	1 (2.0)	[76]
	The Power of YOU-AOD and health program	1 (2.0)	[64]
	Casa-care health service	1 (2.0)	[77]
	**Total**	**5 (10.2)**	
**Refugee and migration status**	Sponsored refugee status	1 (2.0)	[58]
	Forced migration status	1 (2.0)	[54]
	**Total**	**2 (4.1)**	
**Mental health**	Mobile phone-based mental health intervention	1 (2.0)	[71]
	Shelter-based mental health clinic	1 (2.0)	[79]
	**Total**	**2 (4.1)**	
**Creative arts intervention**	Arts-based program	1 (2.0)	[90]
	Hip Hop Self-Expression group intervention	1 (2.0)	[74]
	**Total**	**2 (4.1)**	
**Income support**	Earned income tax credit	1 (2.0)	[53]
	**Total**	**1** (2.0)	
**Group intervention**	Men’s group	1 (2.0)	[52]
	**Total**	**1** (2.0)	
	**TOTAL STUDIES**	**49 (100.0)**	

**Table 5:** Housing First.

A Note on Housing First
Housing First was developed in the 1990s and is a critical policy response in the USA, the UK, Canada, and Europe [101]. It has been suggested that Housing First should be adopted across a community’s entire homelessness response system [102]. Tsembis [103] described Housing First as being two services, housing and support, underpinned by a program philosophy and values [103]. Generally, the model incorporates five broad principles [104], described below by Rhatigan and Blay [104]: 1. Housing—Immediate access to housing with no readiness conditions; 2. Choice—Consumer choice and self-determination; 3. Recover—Recovery orientation; 4. Support—Individualized and person-driven supports; and, 5. Community—Social and community integration. Housing First approaches comprise initiatives that provide housing, either scattered-site or communal/single site implementation [103], with additional “wrap-around” support, for example, to those with mental health and other health conditions as immediate priorities, “without requiring participation in treatment or sobriety as preconditions” [82].

**Table 6:** Classification of cultural and linguistic diversity and related identities.

Classification	No. of Studies (%)	Citation
Ethnicity	10 (20.4)	[60,62,64,72,80,88,89,92,100]
Race/Ethnicity	9 (18.4)	[53,63,65,66,67,68,70,76,78]
Race	5 (10.2)	[56,59,61,69,75]
Ethno-racial status	4 (8.2)	[82,84,86,99]
Ethnicity or Cultural identity; Country of birth	3 (6.1)	[87,93,97]
Migration status; Country of birth/origin.	3 (6.1)	[52,54,58]
No formal classification used	3 (6.1)	[55,57,74]
Race and Ethnicity	2 (4.1)	[71,79]
Ethnicity; Country of birth.	2 (4.1)	[91,95]
Ethno-cultural identity	1 (2.0)	[83]
Racial-ethnic minority status	1 (2.0)	[81]
Ethnic/Cultural background	1 (2.0)	[90]
Ethno-racial minority status	1 (2.0)	[98]
Visible minority; Country of birth	1 (2.0)	[85]
Race/Ethnicity; Country of birth	1 (2.0)	[94]
Ethno-racial status; Country of birth	1 (2.0)	[96]
Race; Latinx status; Ethnic group	1 (2.0)	[77]
**TOTAL**	**49 (100)**	

**Table 7:** Themes and sub-themes.

Theme	Sub-Themes
**Housing provision**	Housing First: efficacy and considerations
	Other service provision models
	Costs of housing models
**Mental health: intersections and interventions**	Housing, homelessness, and mental health
	Mental health interventions for people experiencing homelessness (non-housing)
**Complexity and needs beyond housing**	Social connection and community
	Shame/stigma/discrimination
	Health and support requirements of homeless populations
	Employment, financial assistance, and income support
**Substance use**	Housing provision and substance use
	Substance use interventions (non-housing)
**Service provider and policy issues**	System change
	Service needs and gaps
**The role of cultural and linguistic diversity**	The value of tailored approaches for people from CaLD backgrounds
	The intersection of ethnicity
**Consumer experience**	Working with people with lived experience
	Consumer choice
	Novel ways of engaging

## Supplementary Materials

The following supporting information can be downloaded at: https://www.mdpi.com/article/10.3390/ijerph192416946/s1, Table S1: Studies included in the scoping review.
