# Supplementary material for: Public Health Interventions to Address Housing and Mental Health amongst Migrants from Culturally and Linguistically Diverse Backgrounds Living in High-Income Countries: A Scoping Review
Source: Int J Environ Res Public Health. 2022 Dec 16;19(24):16946. doi: 10.3390/ijerph192416946 (PMC9778908; doi:10.3390/ijerph192416946)
Supplement: Supplementary file 1 [file ijerph-19-16946-s001.zip › ijerph-2044536-supplementary.pdf]

Table S1: Studies included in the scoping review

Author/ Year/ Country	Aim	Participants/Sample size (SS)/Response rate (RR)	Cultural and Linguistic categorization	Evaluation/Research Design/Methods	Intervention type/Duration	Outcome measures
Adair et al. (2017) [80]<br><br>Canada	Examine predictors of outcome trajectories in terms of housing stability for people in Housing First.	Participants: Adults 18 years or older, absolutely or precariously housed, with mental health and/or substance use disorder.<br>SS: 2140<br>RR: 84% (24 months).	Ethnicity.	Study type: Randomized controlled trial.<br>Data collection: Baseline; 3 monthly.<br>Collection method: Interview.<br>Ethical approval: Yes	Intervention: Housing First.<br>Duration: 24-months.	Primary outcome: Housing stability.<br>Predictive factors: Mental health, childhood trauma, self-esteem/ self-efficacy, cognitive impairment.
Aubry et al. (2015) [81]<br><br>Canada	Explore efficacy of Housing First with assertive community treatment.	Participants: Adults with mental illness experiencing homelessness.<br>SS: 950.<br>RR: 90% (12-months).	Racial-ethnic minority status.	Study type: Randomized control trial.<br>Data collection: Baseline; 3 monthly (housing) 6- and 12-months.<br>Collection method: Interviews; housing history.<br>Ethical approval: Yes	Intervention: Housing First.<br>Duration: 12-months.	Housing stability, quality of life, psychiatric symptoms; substance use; community functioning.
Aubry et al. (2016) [82]<br><br>Canada	Explore longer-term efficacy of Housing First with assertive community treatment.	Participants: Adults with mental illness experiencing homelessness.<br>SS: 950.<br>RR: 82%.	Ethno-racial status.	Study type: Randomized controlled trial.<br>Data collection: Baseline; 6-monthly for 21 or 24 months.<br>Collection method: Interviews.<br>Ethical approval: Yes	Intervention: Housing First.<br>Duration: 24-month.	Primary outcomes: housing stability, community functioning.<br>Secondary outcomes: health status, mental health status, quality of life and integration, substance use, hospitalisations, emergency department attendances, arrests.
Aubry et al. (2019) [83]<br><br>Canada	Examine effectiveness of Housing First programs with assertive community treatment for people experiencing serious psychiatric conditions.	Participants: Adults 18 years or older; experiencing homelessness and mental illness.<br>SS: 201<br>RR: 84%	Ethno-cultural identity.	Study type: Randomized control trial.<br>Data collection: Telephone interviews at 3, 9, 15 and 21 months; In-person interviews at 6, 12, 18 and 21 or 24 months.<br>Ethical approval: Yes	Intervention: Housing First with assertive community treatment.<br>Duration: 24-months.	Primary outcomes: Housing stability, community functioning.<br>Secondary outcomes: health measures, quality of life, community integration, substance use, recovery.
Collins et al. (2013) [65]<br><br>United States	Explore accommodation retention in a Housing First program for people experiencing long-term homelessness with severe alcohol problems.	Participants: People experiencing chronic homelessness and severe alcohol problems from a Housing First program.<br>SS: 111.<br>RR: 46%	Race/Ethnicity.	Study type: Non-randomized control trial.<br>Data collection: Baseline; across 2 years.<br>Collection method: self-report measures, housing status.<br>Ethical approval: Yes	Intervention: Housing First single-site.<br>Duration: 24-months.	Interest in housing, housing retention, substance use, mental health, physical health, demographic characteristics.
Dwyer & Brown (2008) [54]<br><br>United Kingdom	Examine issues related to housing and susceptibility of dispersed forced migrants to homelessness.	Participants: Dispersed forced migrants, living in the UK.<br>SS: 34<br>RR: 100%.	Migration status; Country of birth.	Study type: Qualitative – no design stated.<br>Collection method: Interviews; Focus groups.<br>Ethical approval: Not recorded.	Intervention: Migration status.	Housing stability, adequacy of government and privately provided housing, standard of accommodation and housing-related support, impact of changes in socio-legal status, risk of homelessness.
Gewitz et al. (2009) [55]<br><br>United States	Explore associations between parenting, child adjustment and parent mental health in formerly homeless families in supportive housing.	Participants: Families with 6–12-year-old children, previously experiencing homelessness, living in supportive housing communities, in two USA cities.<br>RR: 100%	Formal classification not used *(Multiracial, minority).*	Study type: Randomized control trial.<br>Collection method: Interview; Self-assessment; Observation.<br>Ethical approval: Yes	Intervention: Supportive housing.	Parental mental health, parenting self-efficacy, parenting practices (various measures), child adjustment.<br>Control variables: child age, parent income, education.
Gilmer et al. (2010) [66]<br><br>United States	Examine the impact of Full-Service Partnerships on recovery, use of mental health services and costs, and quality of life.	Participants: Adults experiencing chronic homelessness living with severe mental Illness.<br>SS: 363.<br>RR: 100%	Race/Ethnicity.	Study type: Quasi-experimental, difference-in-difference design.<br>Ethical approval: Yes	Intervention: Full-service Partnership (partly funded permanent housing and support services).<br>Duration: 32-months.	Recovery outcomes, health, mental health service utilization, justice system use, associated expenses.
Gilmer et al. (2014) [67]<br><br>United States	Examine impact of Full-Service Partnerships on health service use and costs, compared with usual care.	Participants: People experiencing serious psychiatric illness and homelessness or at risk of homelessness.<br>SS: 20,462.<br>RR: not reported.	Race-Ethnicity.	Study type: Quasi-experimental, pre-post, intent-to-treat design.<br>Collection method: Data from the Client and Services Information system; Medi-Cal Short Doyle; Inpatient Fee-for-Service Consolidation.<br>Ethical approval: Yes	Intervention: Full-service Partnerships (partly funded permanent housing and support services).	Mental health service utilization and costs
Gulcur et al. (2003) [56]<br><br>United States	Compare outcomes of Housing First and Continuum of Care for people with serious mental illnesses and dual diagnoses.	Participants: Street present groups, who were in psychiatric hospitals prior to study entry.<br>SS: 225.<br>RR: 96% (6 months), 90% (24 months).	Race.	Study type: Randomized control trial.<br>Data collection: Baseline; 6-monthly.<br>Collection method: Interviews.<br>Ethical approval: Yes	Intervention: Housing First.<br>Duration: 24-months.	Residential location, accommodation costs.
Guo et al. (2016) [68]<br><br>United States	Document mental health outcomes associated with Ecologically Based Treatment.	Participants: Women experiencing homelessness, caring for a biological child aged 2–6 years, with substance abuse or dependence disorder.<br>SS: 60.<br>RR: 100% (Ecologically Based Treatment group), 80% (usual service provision group).	Race/Ethnicity.	Study type: Randomized control trial.<br>Data collection: Baseline; 3, 6, 9-months post.<br>Collection method: Range of forms.<br>Ethical approval: Yes	Intervention: Ecologically Based Treatment.<br>Duration: 9-months.	Substance use and dependence, physical health, mental health, self-efficacy, intimate partner violence, child behaviours.
Hanrahan et al. (2005) [57]<br><br>United States	Describe qualities of mothers experiencing homelessness participating in the Mothers' Project and program achievements.	Participants: Mothers with a history of homelessness and mental illness, participating in the Thresholds' Mothers' Project from 1996-2002.<br>SS: 24.<br>RR: 92% (6 months), 79% (12 months).	Formal classification not used (*European-American, Hispanic*)	Study type: Retrospective chart review.<br>Data collection: intake; 6, 12 months post.<br>Ethical approval: Not recorded.	Intervention: Thresholds' Mothers' Project (psychosocial rehabilitation and intensive care management, mental and physical health services, primary intervention services for children).<br>Duration: 12-months.	Primary outcome: Residential status.<br>Other outcomes: hospital stays, judicial outcomes, substance use, child progress, custody.

Author/ Year/ Country	Aim	Participants/Sample size (SS)/Response rate (RR)	Cultural and Linguistic categorization	Evaluation/Research Design/Methods	Intervention type/Duration	Outcome measures
Harris et al. (2019) [69]<br><br>United States	Examine impact of supportive housing models and neighbourhoods on social integration outcome.	Participants: Supportive housing residents, 39 years old or older, English or Spanish speaking, currently without a home, without minor children.<br>SS: 405.<br>RR: 100%	Race.	Study type: Longitudinal.<br>Data collection: Baseline; 3-months post.<br>Collection method: Questionnaire; Interviews.<br>Ethical approval: Yes	Intervention: Permanent Supportive Housing.<br>Duration: 12-months.	Housing model, neighbourhood, social networks, homelessness, mental health/ health/ substance use, demographics.
Hwang et al. (2012) [84]<br><br>Canada	Explore and describe recruitment and engagement people involved in the At Home/ Chez Soi Housing First intervention.	Participants: Adults experiencing homelessness and serious psychiatric illness, living in the Toronto area.<br>SS: 575.<br>RR: not reported.	Ethno-racial identity.	Study type: Randomized control trial.<br>Data collection: Baseline<br>Collection method: Questionnaires.<br>Ethical approval: Yes	Intervention: Housing First.<br>Duration: 48-months.	Age, gender, country of birth, language of origin, ethnic or cultural identity, marital status, number of children, homelessness status, education, employment, mental health status, functional and cognitive impairment, referral source.
Karim et al. (2016) [100]<br><br>United Kingdom	Understand mental health issues and parenting challenges in homeless families when first becoming homeless.	Participants: Families, experiencing homelessness, in two homeless shelters.<br>SS: 81 families.<br>RR: 43%.	Ethnic status.	Study type: Longitudinal.<br>Data collection: Baseline; 4 months post.<br>Collection method: Quantitative tools; Interviews.<br>Ethical approval: Not recorded.	Intervention: Rehousing in the community.<br>Duration: 4-months.	Primary outcomes: parent and child mental health measures.<br>Parenting problems, and service satisfaction.
Kirst et al. (2015) [85]<br><br>Canada	Explore impact of scatter-site Housing First on substance use outcomes.	Participants: Adults aged 18 or older, experiencing homelessness or housing precarity, living with a mental health issue with or without substance use.<br>SS: 575.<br>RR: 80% (24 months).	Visible minority; Country of birth.	Randomized control design.<br>Data collection: Baseline; 6, 12, 18, 24 months.<br>Ethical approval: Yes	Intervention: Housing First.<br>Duration: 24-months.	Primary outcome: Substance use.<br>Gender, age, minority status, country of birth, marital status, duration of homelessness, employment, education, chronic conditions, number of children.
Mejia-Lancheros et al. (2020) [86]<br><br>Canada	Identify trajectories in relation to stigma and discrimination and mental health in homeless adults, and the effect of Housing First.	Participants: Adults, experiencing homelessness, taking part in the Toronto site of the AHCS Housing First study.<br>SS: 414.<br>RR: 99.0% (stigma trajectory), 97.6% (discrimination trajectory).	Ethno-racial status.	Study type: Randomized control trial.<br>Data collection: Baseline; 1, 2-years post.<br>Collection method: Interviews.<br>Ethical approval: Yes	Intervention: Housing First.<br>Duration: 24-months.	Discrimination: The 'Unfair treatment" subscale of the Discrimination and Stigma Scale (DISC-12).<br>Stigma: 10-item Stigma Experiences Scale.<br>Mental health, substance use measures: discrimination in health scenarios because of health problems.
Murdie (2008) [58]<br><br>Canada	Assess whether more difficulty in accessing accommodation is experienced in refugee claimants or sponsored refugees.	Participants: Sponsored refugees and refugee claimants in Toronto, Canada; Key informants.<br>SS: Exact numbers not provided.<br>RR: not reported.	Refugee status; Country of birth	Study type: Qualitative – specific design not stated.<br>Collection method: Interviews.<br>Ethical approval: Not recorded.	Intervention: Sponsored refugee experience compared to refugee claimant experience.	Housing trajectories.<br>Initial housing experience, search for permanent accommodation, other accommodation outcomes (tenure, property type, rent), income, employment status.
O'Campo et al. (2016) [87]<br><br>Canada	Examine how Housing First creates changes in housing stability, justice system involvement, healthcare access and health outcomes in people experiencing homelessness and mental illness.	Participants: Adults experiencing homelessness and psychiatric conditions.<br>SS: 197.<br>RR: not reported.	Ethnicity or cultural identity; Country of birth	Study type: Randomized trial.<br>Data collection: 3-monthly.<br>Collection method: Interviews; Questionnaires.<br>Ethical approval: Yes	Intervention: Housing First with assertive community treatment.<br>Duration: 24-months.	Primary outcome: Housing stability.<br>Secondary outcomes: quality of life, functional ability.<br>Exploratory outcomes: mental health symptom severity, substance use, community integration, health service and justice use.
Padgett, et al. (2006) [59]<br><br>United States	Assess outcomes related to substance use and treatment in two groups - Housing First and treatment first.	Participants: People experiencing homelessness in New York City, with severe mental illness.<br>SS: 225.<br>RR: 35% (at 12 months).	Race.	Study type: Longitudinal experimental study.<br>Data collection: 6-monthly for 48 months.<br>Collection method: Interviews.<br>Ethical approval: Yes	Intervention: Housing First and treatment first.<br>Duration: 48-months.	Use of alcohol and other drugs, engagement in with treatment interventions.
Padgett et al. (2011) [70]<br><br>United States	Compare differences in substance use and treatment utilisation amongst individuals in either Housing First or Treatment First programs.	Participants: Adults experiencing homelessness, entering either program, with a DSM Axis-1, and history of substance use.<br>SS: 83.<br>RR: 90%	Race/Ethnicity.	Study type: Qualitative.<br>Data collection: at 0-, 6-, 12-months; monthly<br>Collection method: Interviews; Phone calls.<br>Ethical approval: Yes	Intervention: Housing First.<br>Duration: 12-months.	Substance use, treatment for substance abuse.
Palepu et al. (2013) [88]<br><br>Canada	Investigate substance dependence and stability of accommodation stability in Housing First adults experiencing homelessness with mental health issues.	Participants: Adults 19 years or older, experiencing homelessness or housing precarity, living with a current mental disorder.<br>SS: 497.<br>RR: 96%.	Ethnicity.	Study type: Randomized control trials.<br>Data collection: Baseline; 3-monthly.<br>Collection method: Interviews.<br>Ethical approval: Yes	Intervention: Housing First.<br>Duration: 12-months.	Primary outcome: Residential stability.<br>Primary independent variable: substance dependency.<br>Mental health symptoms and severities.
Patterson et al. (2013) [89]<br><br>Canada	Explore perceived quality of life in adults experiencing homelessness and mental health issues after receiving housing models.	Participants: Adults 19 years or older, experiencing homelessness or housing precarity, living with a current mental disorder.<br>SS: 497.<br>RR: 95%.	Ethnicity.	Study type: Randomized control trials.<br>Data collection: Baseline; 2, 6 months post.<br>Collection method: Questionnaires.<br>Ethical approval: Yes	Intervention: Housing First.<br>Duration: 12-months.	Primary outcomes: Quality of life.<br>Other measures: mental illness, medical conditions, infectious disease, demographics.
Pilkauskas & Micelmore (2019) [53]<br><br>United States	Understand how Earned Income Tax Credits impact on housing and living circumstances of single mothers.	Participants: Single mothers in the USA receiving Earned Income Tax Credits.<br>SS: 853,012.<br>RR: 100%	Race/Ethnicity.	Study type: Quasi-experimental study.<br>Collection method: Data from the Current Population Survey, the American Community Survey/decennial census, and the Fragile Families and Child Wellbeing Survey.<br>Ethical approval: Not recorded	Intervention: Earned Income Tax Credit.	Homelessness and eviction, cost burden, housing arrangements.

Author/ Year/ Country	Aim	Participants/Sample size (SS)/Response rate (RR)	Cultural and Linguistic categorization	Evaluation/Research Design/Methods	Intervention type/Duration	Outcome measures
Schueller et al. (2019) [71]<br><br>United States	Determine feasibility, acceptability, and effects of a multicomponent mobile phone–based program for homeless adults.	Participants: English-speaking, young adults, aged between 18 and 24, currently experiencing homelessness and sleeping in a Chicago-based homeless shelter.<br>SS: 35.<br>RR: 66%.	Race and Ethnicity.	Study type: Feasibility pilot trial.<br>Data collection: Baseline; 4 weeks.<br>Collection method: Self-report assessments.<br>Ethical approval: Yes	Intervention: Mobile phone- based program for young adults.<br>Duration: 1 month.	Feasibility, acceptability, mental health symptoms and self-reported changes in depression, PTSD, and emotional regulation.
Schutt et al. (2009) [60]<br><br>United States	Explore impact of housing, supports, and individual characteristics on housing loss among formerly homeless people with mental health conditions.	Participants: Adults, experiencing homelessness and mental illness, across various sites in U.S.<br>SS: 894.<br>RR: 79%.	Ethnicity.	Study type: Randomized control trials.<br>Data collection: 18 month follow up.<br>Collection method: Interviews.<br>Ethical approval: Not recorded.	Intervention: rental subsidy vouchers plus active community treatment or comprehensive case management; psychiatric rehabilitation services including outreach and housing respite; community housing with or without intensive treatment; and clinical case management with either group housing or individual apartments.<br>Duration: 12 – 24 months.	Housing outcomes, lifetime substance use, symptoms of mental illness.
Schwan et al. (2018) [90]<br><br>Canada	Explore impacts of an art-based program in the lives of young people experiencing homeless.	Participants: Young people, aged 16-24, experiencing homelessness; Program staff.<br>SS: 23.<br>RR: 100%.	Ethnic/Cultural background.	Study type: Grounded theory.<br>Collection method: Interviews.<br>Ethical approval: Yes	Intervention: Arts-based programming at a homeless shelter.<br>Duration: 5-months.	Young people's conceptions of the value, and significance of art creation for them, the impacts attributed to artmaking and art program participation, and elements of the program viewed as effective.
Slesnick & Erdem (2013) [72]<br><br>United States	Test impact of Ecologically Based Treatment for mothers facing homelessness and substance use, whilst caring for children.	Participants: Mothers experiencing homelessness, with children in their care.<br>SS: 60.<br>RR: 100% (intervention group).	Ethnicity.	Study type: Randomized control trial.<br>Data collection: Baseline; 3-, 6- 9-months post.<br>Collection method: Range of forms.<br>Ethical approval: Yes	Intervention: Ecologically Based Treatment.<br>Duration: 9-months.	Substance use, housing stability.
Slesnick et al. (2015) [73]<br><br>United States	Compare treatment outcomes for young people with substance use disorder between three different intervention approaches.	Participants: Young people experiencing homelessness and substance use disorder, living in Ohio.<br>SS: 270.<br>RR: 75% (3 months), 76% (12 months).	Ethnicity.	Randomized control trial.<br>Ethical approval: Yes	Intervention: Community reinforcement, motivational enhancement therapy and case management.<br>Duration: 12-months.	Primary outcomes: substance use including alcohol.<br>Secondary outcomes: depression symptoms, internalising/externalising problems, victimisation, homelessness, coping.
Somers et al (2013) [91]<br><br>Canada	Describe recruitment of two samples of people experiencing homelessness and mental illness with different complexity of needs.	Participants: Adults 19 years or older, experiencing homelessness or housing precarity, living in Vancouver, with a current mental disorder.<br>SS: 497.<br>RR: 97%.	Ethnicity; Country of birth.	Study type: Randomized control trials.<br>Data collection: 3-monthly for 2 years,<br>Collection method: Interviews; Administrative data.<br>Ethical approval: Yes	Intervention: Housing First.<br>Duration: 24-months.	Housing and vocational status, mental and physical health, community integration and functioning, quality of life, level of community service use.
Somers et al. (2015) [92]<br><br>Canada	Compare daily substance use in Housing First compared to usual service provision.	Participants: Adults 19 years or older, experiencing homelessness or housing precarity, living in Vancouver, with a current mental disorder.<br>SS: 497.<br>RR: 90.1% (12 months), 79.3% (24 months).	Ethnicity.	Study type: Randomized control trials.<br>Data collection: Baseline; 6-monthly.<br>Collection method: Interviews.<br>Ethical approval: Yes	Intervention: Housing First.<br>Duration: 24-months.	Primary outcome: Daily substance use.<br>Demographics, homelessness history, psychiatric diagnosis, symptom severity, comorbidity, duration of stable housing.
Stefanic & Tsemberis (2007) [61]<br><br>United States	Document housing retention and status and cost-effectiveness of Housing First programs for chronically homeless people with mental health problems.	Participants: People with mental illness using shelters on a long-term basis.<br>SS: 260<br>RR: Not reported.	Race.	Study type: Randomized control trial.<br>Collection method: Data from administrative records from a government agency and Housing First agencies.<br>Ethical approval: Not recorded.	Intervention: Housing First, provided by two different agencies.<br>Duration: 48-months.	Housing status, housing retention, housing service costs.
Stergiopoulos et al. (2012) [97]<br><br>Canada	Present early implementation and fidelity evaluation of a novel Housing First Ethno-Racial Intensive Case Management model, designed to meet needs of homeless people with mental illness from ethno-racial groups.	Participants: People from a defined ethno-racial group taking part in the At Home/Chez Soi Research Demonstration Project in Homelessness and Mental Health.<br>SS: 204 (intervention), 93 (evaluation)<br>RR: Not reported.	Ethnic or cultural identity; Country of birth	Study type: Randomized control trial.<br>Collection method: Interviews; Focus groups; Observation; Document review.<br>Ethical approval: Yes	Intervention: Housing First Ethno-Racial Intensive Case Management model.	Demographics, mental illness diagnoses and extent of community functioning.<br>Fidelity: 1) housing choice and structure; 2) separation of housing and services; 3) service philosophy; 4) service array; and 5) program structure.
Stergiopoulos et al. (2015) [93]<br><br>Canada	Evaluate effectiveness of Housing First with Intensive Case Management amongst ethnically diverse adults experiencing homelessness.	Participants: Adults experiencing homelessness or housing precarity and mental illness, living in Toronto.<br>SS: 378.<br>RR: 92% (12 months), 85% (24-months).	Ethnic or cultural identity; Country of birth	Study type: Randomized control trial.<br>Data collection: 3-monthly.<br>Collection method: Interviews.<br>Ethical approval: Yes	Intervention: Housing First with Intensive Case Management.<br>Duration: 24-month duration.	Primary outcome: Housing stability.<br>Secondary outcomes: health measures, social functioning and quality of life, health service use.
Stergiopoulos et al. (2015a) [94]<br><br>Canada	Investigate impact of scattered-site housing with intensive case management on housing stability and other measures for adults experiencing homelessness and mental health issues with moderate support needs.	Participants: Adults experiencing homelessness and mental illness with moderate support needs, with or without substance use.<br>SS: 1198.<br>RR: 92.2% (12 months), 84.8% (24 months).	Country of birth; Race/Ethnicity.	Study type: Randomized trial.<br>Data collection: 3-monthly.<br>Collection method: Interviews.<br>Ethical approval: Yes	Intervention: Scattered-site housing and off-site intensive case management services.<br>Duration: 24-months.	Primary outcome: housing stability.<br>Secondary outcome: generic quality of life.
Stergiopoulos et al. (2016) [95]	Determine effectiveness of a Housing First program with anti-racism and anti-oppression practices for adults experiencing homelessness and mental	Participants: Adults from ethnic minorities, experiencing homelessness and mental health issues, living in Toronto.<br>SS: 237.<br>RR: 90% (12 months), 79% (24 months).	Ethnicity; Country of birth.	Study type: Randomized control trial.<br>Data collection: 3-month intervals; at 6, 12, 18, 24 months.<br>Collection method: Interviews.	Intervention: Housing First enhanced with anti-racism and anti-oppression practices.<br>Duration: 24-months.	Primary outcome: Housing stability.<br>Secondary outcomes: health measures, social measures, services utilization.

Author/ Year/ Country	Aim	Participants/Sample size (SS)/Response rate (RR)	Cultural and Linguistic categorization	Evaluation/Research Design/Methods	Intervention type/Duration	Outcome measures
Canada	health issues from diverse ethnic minority backgrounds.			Ethical approval: Yes		
Stergiopoulos et al. (2019) [96]<br><br>Canada	Evaluate impact of Housing First on accommodation and health outcomes for people experiencing homelessness and mental health issues.	Participants: People experiencing homelessness and mental health issues, with or without substance use disorder.<br>SS: 378.<br>RR: 81% (phase 1); 88% (phase 2).	Country of birth; Ethno-racial status.	Study type: Randomized control trial.<br>Data collection: 3, 6 and 12 months.<br>Collection method: Interviews.<br>Ethical approval: Yes	Intervention: Housing First.<br>Duration: 72-months.	Primary outcome: Housing stability.<br>Secondary outcomes: quality of life, (e.g. community functioning, substance use).
Thompson et al. (2017) [52]<br><br>United Kingdom	Describe intervention with men residing in London from the Horn of Africa experiencing psychosocial issues.	Participants: Men from the Horn of Africa, experiencing homelessness, living in London.<br>SS: 20.<br>RR: 9.	Country of origin; refugee status.	Study type: Interventional study design.<br>Collection method: Observational data.<br>Ethical approval: Not recorded.	Intervention: Men's group with a mental health and housing focus.<br>Duration: 8-weeks.	Attendance; group interactions; perspectives of participants on housing issues, group logistics and benefits.
Travis et al. (2019) [74]<br><br>United States	Explore effect of a Hip-Hop based program in adults experiencing homelessness and severe mental illness in a shelter setting.	Participants: Adults experiencing homelessness and severe mental illness in a shelter setting.<br>SS: Not reported. Study site area had a population of people experiencing homelessness of approximately 50,000 - 100,000<br>RR: not stated.	No classification specified.	Study type: Qualitative - design not formally identified.<br>Collection method: Observation; Document analysis.<br>Ethical approval: Yes	Intervention: Hip Hop Self-Expression group. Group based pilot program, built on the MUZUZE Hip Hop and empowerment framework.	Primary outcomes: Social- emotional learning indicators (self-awareness and social awareness).<br>Secondary outcomes: empowerment- based positive youth development indicators (e.g., connection and sense of community).
Tsai et al. (2012) [75]<br><br>United States	Examine if supported housing leads to improvements in social integration for adults experiencing homelessness.	Participants: Adults experiencing homelessness and mental health issues, participating in the Collaborative Initiative to Help End Chronic Homelessness.<br>SS: 550.<br>RR: 73%.	Race.	Study type: Longitudinal.<br>Data collection: Baseline; 6, 12-month post.<br>Collection method: Quantitative data (survey items).<br>Ethical approval: Not recorded.	Intervention: Collaborative Initiative to Help End Homelessness.<br>Duration: 12-months.	Primary outcome: social integration (housing, work, social support, community participation, civic activity, religious faith). Clinical status (mental disorder; substance use disorder).
Tsemberis & Eisenberg (2000) [62]<br><br>United States	Explore impact of the Pathways to Housing supported housing program.	Participants: People with mental illness experiencing homelessness, living on the street.<br>SS: 1841.<br>RR: not reported.	Ethnicity.	Study type: Comparative study.<br>Collection method: Record of housing tenure.<br>Ethical approval: Not recorded.	Intervention: Pathways to Housing.<br>Duration: 57-months.	Housing status, mental health symptoms, alcohol and substance use, social networks, housing satisfaction.
Tsemberis et al. (2004) [63]<br><br>United States	Explore impacts of Housing First for people experiencing homelessness and psychiatric illness on a range of outcomes related to choice, mental health, and housing.	Participants: Individuals with mental illness experiencing homelessness who had spent at least half of their time on the street or in shelters in the almost 30 days.<br>SS: 225.<br>RR: 88% (6 months), 78% (24 months).	Race/Ethnicity.	Study type: Randomized control trial.<br>Data collection: 6-monthly for 24 months.<br>Collection method: Interview.<br>Ethical approval: Yes	Intervention: Housing First.<br>Duration: 24-months.	Consumer choice, stability of housing, substance use and interventions, mental health symptoms.
Tucker et al. (2017) [76]<br><br>United States	Evaluate effectiveness of a group-based motivational interviewing program on alcohol and substance use and sexual health practices among people experiencing homelessness.	Participants: Young adults, aged 18-25 experiencing homelessness, in Los Angeles County, seeking services at one of two drop-in centre study sites.<br>SS: 200.<br>RR: 48% (full program), 79% (multiple sessions).	Race/Ethnicity.	Study type: Randomized control trial.<br>Data collection: Baseline; 3 months post.<br>Collection method: Surveys.<br>Ethical approval: Not recorded.	Intervention: AWARE: a group-centered Motivational Interviewing program.<br>Duration: 16-weeks.	Alcohol and substance use-related outcomes, sexual health-related outcomes, fidelity, acceptability measures.
Urbanoski et al. (2017) [98]<br><br>Canada	Determine whether substance use disorders modify the effectiveness of a Housing First intervention.	Participants: Adults experiencing homelessness or housing precarity, with a mental disorder, living in Canada.<br>SS: 2255.<br>RR: 95.5%	Ethno-racial minority status.	Study type: Randomized control trial.<br>Data collection: Baseline; 6-monthly for 21 or 24 months.<br>Collection method: Interviews.<br>Ethical approval: Yes.	Intervention: Housing First with Intensive Case Management or assertive community treatment.<br>Duration:21-months.	Primary outcomes: days in housing, community functioning.<br>Secondary outcomes: quality of life, mental health symptoms.
Volk et al. (2016) [99]<br><br>Canada	Identify characteristics of people who experience housing instability at 12 months post- entering Housing First.	Participants: Adults experiencing homelessness and mental illness.<br>SS: 1162.<br>RR: 100%.	Ethno-racial status.	Study type: Randomized controlled trial.<br>Data collection: 3-monthly for 12 months.<br>Collection method: Interview; housing stability data.<br>Ethical approval: Yes.	Intervention: Housing First.<br>Duration: 24-months.	Primary outcome: Housing stability.<br>Predictors: community functioning, mental disorders, community psychological integration, quality of life, healthcare use, social services use, justice system use.
Walter et al. (2019) [77]<br><br>United States	Assess changes in health and health care utilisation amongst Latinx adults with mental illness and substance use taking part in integrated behavioural and primary health care.	Participants: Latinx adults experiencing homelessness, living in the USA.<br>SS: 107.<br>RR: 100%	Race; Latinx status; Ethnic group.	Study type: Longitudinal.<br>Data collection: Baseline; 6, 12 months.<br>Collection method: Interviews.<br>Ethical approval: Yes.	Intervention: CasaCare (Person-Centered Health Home) - culturally tailored medical, behavioural and wellness supports, services to address social determinants of health.<br>Duration: 12-months.	Primary outcomes: depression and anxiety, substance use.<br>Secondary outcomes: healthcare utilization, homelessness status.
Wenzel et al. (2009) [64]<br><br>United States	Address co-occurrence of alcohol and other drug use, HIV risk behaviours, and intimate partner violence in women experiencing homelessness.	Participants: Women experiencing homelessness, aged 18-25, in Los Angeles County, who spoke/understood English.<br>SS: 31.<br>RR: 87%.	Ethnicity.	Study type: Pilot group program.<br>Collection method: Questionnaires; Focus groups; Feedback sessions.<br>Ethical approval: Yes.	Intervention: The Power of YOU: group addressing alcohol, substance use, HIV risk behaviours, partner violence.<br>Duration: 5-months.	Alcohol intoxication, number of male sex-partners, intimate partner violence. Satisfaction, enjoyment of sessions.
Wenzel et al. (2019) [78]<br><br>United States	Investigate experiences of discrimination in individuals who moved into permanent supportive housing.	Participants: Adults experiencing homelessness, moving to permanent supportive housing.<br>SS: 421.<br>RR: 90.9%.	Race/Ethnicity.	Study type: Longitudinal.<br>Data collection: Before/within 5 days of move-in; 3, 6, 12 months after moving in.<br>Collection method: Interviews.<br>Ethical approval: Yes.	Intervention: Permanent supportive housing.<br>Duration: 12-months.	Experiences of discrimination, perceived reasons for discrimination.
Winiarski et al. (2020) [79]<br><br>United States	Describe development of shelter-based clinical model for youth experiencing homelessness and evaluate its outcomes on service utilization.	Participants: Young people experiencing homelessness, who attended a shelter-based clinic in a Midwestern city.<br>SS: 77.<br>RR: 49.4% (first session), 13% (second session).	Race and Ethnicity.	Study type: Cross-sectional evaluation.<br>Data collection: Intake.<br>Collection method: Clinician rating forms.<br>Ethical approval: Yes.	Intervention: Shelter-based mental health clinic involving free psychology and psychiatry sessions.<br>Duration: 24-months.	Mental health service utilization, mental health status of homeless youth.
